# The Educational Value of Outdoor Games

**Published:** 1890-05

**Authors:** 


### THE EDUCATIONAL VALUE OF OUTDOOR GAMES.

Organized play produces a most useful effect. It trains the boy to do correctly just what he is told to do, and, while his spontaneous action is encouraged, he is kept ever ready to act according to circumstances. Play is healthful; so is the alternation of mental work and active play. In mental action, the brain centers probably act much in stimulating one another; in play, the muscles are stimulated by the brain centers, and the purely mental action is diminished. Thus, play is not merely muscular exercise, but a change in the kind of brain action, and probably of the action of the special centers.

Even if the fact were not well established, the physiologist would expect to find that moderate athletics and success in mental work are not divorced from one another. This is well illustrated in the list of scholarships recently gained by the boys of St. Paul's school; all the athletic leaders are named in the list. The tendency to self contemplation, also, which is engendered by the modern system of competitive examinations, is to some extent counteracted by athletics. In the examination, the individual wins, not his class; in the cricket club, the eleven wins, though one individual may make the winning score.

It is sometimes said that athletics make good bodies to the neglect of mental culture. That may be true when too much time and attention are given to the river and the cricket-field; but it should be well understood that a highly organized game does exercise the brain as well as the muscles, though not in exactly the same way, nor probably in the same parts, as does so called intellectual training. A just balance between play and work may be struck for the individual, by noting what duration of mental exercise can be borne without the signs of fatigue following. Recreation of the athletic kind is most useful in turning "the brain overpressed with thoughts," to other modes of action, and preventing it from continuously acting in mental modes, producing a cloud of uncontrolled thoughts, to be followed by troubled sleep and dreams. Habits of bodily activity are often the best cure for sickly states of mind.—*British Medical Journal.*

